# Relative Expression of OCT4, SOX2 and NANOG in Oral Squamous Cell Carcinoma Versus Adjacent Non- Tumor Tissue

**DOI:** 10.31557/APJCP.2019.20.6.1649

**Published:** 2019

**Authors:** Fereshteh Baghai Naini, Pouyan Amini Shakib, Alireza Abdollahi, Mahshid Hodjat, Hadiseh Mohammadpour, Neda Kardouni Khoozestani

**Affiliations:** 1 *Department of Oral and Maxillofacial Pathology, Faculty of Dentistry, *; 2 *Dental Research Center, Dentistry Research Institute, *; 3 *Department of Pathology, Imam Khomeini Hospital Complex, *; 4 *Iran National Tumor Bank, Cancer Institute, Imam Khomeini Hospital Complex, Tehran University of Medical Science, Tehran, Iran. *

**Keywords:** OCT4, SOX2, NANOG, squamous cell carcinoma, gene expression

## Abstract

**Objective:**

Oral squamous cell carcinoma (OSCC) accounts for over 90% of oral neoplasms. Finding molecular markers for predicting prognosis is a high priority. The core transcription factors, OCT4, SOX2, and NANOG that regulate embryonic stem cell pluripotency have been implicated in progression of various malignancies. The predictive value of these markers and their role in the development of OSCC is still controversial. In this study, we therefore evaluated their expression in OSCCs and adjacent non-tumor tissue.

**Methods:**

A total of 60 frozen tumor and adjacent non-tumor tissue samples from 30 patients with OSCC were examined using quantitative reverse transcription polymerase chain reaction (qRT-PCR). Clinical and pathological data of patients including tumor stage, lymph node metastasis and tumor grade were also recorded.

**Results:**

Expression of SOX2 was significantly higher in adjacent non-tumor as compared to tumor tissue (P=0.04). No statistically significant differences were found for expression of OCT4 (P=0.50) and NANOG (P=0.68). Also, there was no significant association between expression of OCT4, SOX2, and NANOG and clinical or pathological data (P>0.05), although slightly higher values were noted in patients without lymph node metastasis.

**Conclusion:**

Based on the present data, decreased expression of SOX2 is correlated with carcinogenesis in the oral cavity and development of OSCC.

## Introduction

It is known that 5% of all tumors develop in the head and neck region, out of which 50% occur in the oral cavity (Geum et al., 2013). The risk of recurrence of tumors in the oral cavity is relatively high compared to the tumors in other parts of the body (Liu et al., 2007). Presently, oral carcinomas are among the 10 most common malignancies in humans and has been reported as the major cause of morbidity, mortality and facial deformity worldwide (Bau et al., 2011; Bernier, 2011). Squamous cell carcinoma (SCC) accounts for over 90% of oral neoplasms (Sharma et al., 2010). The incidence of neck metastasis in oral cancer ranges from 19 to 34% and evidence shows that patients with neck metastasis have a poorer prognosis (Liu et al., 2007). Despite recent advances in cancer treatment, the rate of morbidity and mortality is still high in a considerable number of patients presenting with local recurrence, systemic metastasis or secondary tumors, which highlights the need for strategies for earlier detection and most effective treatment of cancer (Woolgar et al., 1995).

Tumors are comprised of subpopulations of cells, some of which display increased tumorigenic properties known as the cancer stem cell (CSC) (Al-Hajj and Clarke, 2004; Allegra et al., 2014; Islam et al., 2015; Reya et al., 2001). The presence of stem cell makers has been previously proven in different malignancies such as leukemia and breast cancer (Fillmore and Kuperwasser, 2008; Griffin and Lowenberg, 1986). The emergence of CSCs and detection of CSC markers in different tumoral tissues have profound impact on the development of molecular treatment modalities and replacing the conventional cancer treatments with more efficient alternative approaches (Al-Hajj and Clarke, 2004). So far, many key CSC markers have been introduced that are involved in the progression and development of OSCC (Al-Hajj and Clarke, 2004; Reya et al., 2001). The regulatory role of CD133 (Yu et al., 2016) and CD44 (Krishnamurthy and Nör, 2012) in self-renewal of CSC and tumor progression have been reported previously. Also studies focused on the core transcription factors OCT4, SOX2 and NANOG as the prognostic CSC markers in OSCC (Chiou et al., 2008; Vaiphei et al., 2014). A significant association has been reported between the expression of OCT4, SOX2, and NANOG with the grade of OSCC (Chiou et al., 2008; Vaiphei et al., 2014). 

OCT4, SOX2 and NANOG are the key transcription factors involved in pluripotency of embryonic stem cells and also regulating the fate of stem cells (Li et al., 2015; Nichols et al., 1998; Ren et al., 2016). Their expression decrease during cells differentiation (Fu et al., 2016; Islam et al., 2015; Ren et al., 2016). They are also play critical role in cellular reprogramming of somatic cells. 

Cancer initiation is the consequence of a multi-stage process of genetic changes, which occur prior to the histopathological occurrence of cancer. It is believed that these molecular changes can be detected even at initial stages when the tissue still appears histologically normal. Therefore, finding the key molecules undergoing changes at the very early stage of carcinogenesis can greatly enhance early detection of OSCC (Allegra et al., 2014; Islam et al., 2015).

This study sought to evaluate the expression of OCT4, SOX2 and NANOG CSC markers in OSCC and non-tumoral adjacent tissue specimens.

## Materials and Methods

This observational study was conducted on biopsy samples taken from the tumoral tissue and adjacent non-tumoral tissue of 30 patients with primary diagnosis of OSCC retrieved from the tumor bank of the Pathology Department of Cancer Institute at Imam Khomeini Hospital. Patients were selected via non-probability convenience sampling. Demographic information of patients including age, sex, and ethnicity, the degree of differentiation of tumor and clinical stage of the tumor based on the recorded information were all collected in a checklist. The study was performed in accordance with the ethics board approval of Imam Khomeini Hospital.

*RNA extraction*

The mRNA was extracted from each specimen for real-time PCR to study the expression of OCT4, SOX2 and NANOG using RNeasy Mini kit (Qiagen, Germany) according to the manufacturer protocol. In brief, frozen tissue at -80°C was cut by a sterile scalpel, diced on a plate and transferred to the homogenizing tube containing 1mL RLT reagent. After shaking for 10 seconds, the lysate was centrifuged (1200rpm,) for 3 min at 4°C and the supernatant was transferred to a new tube containing 1mL of 70% ethanol. Next, the mixture was passed through the RNeasy spin column followed by 2 washing step using 700 μL RW1 and 500μL of RPE buffer. Finally, 30-50μL RNase-free water was added to the spin column and after centrifugation, the isolated RNA was frozen at -80°C.

For assessing the RNA integrity, 1 to 2μL of the extracted RNA was subjected to 1.5% agarose gel electrophoresis with 142V voltage for 15 min and the 28S and 18S ribosomal RNA bands were visualized by staining with EtdBr. Also, the purity of the extracted RNA was determined via the absorbance ratio of 260 nm/280nm using spectrophotometer (BioTek, USA).

*cDNA synthesis*

synthesis of cDNA was done using Scientific RevertAid First Strand cDNA Synthesis kit (Thermo, UK). Briefly, the extracted total RNA (5-10ng) were mixed with 1-2μL random hexamer primers and DEPC-treated water up to10μL. Next, the solution was heated at 65°C for 5 min and chilled at 4°C. 10μL of the RT-PreMix (2x) was added to the solution and stored at 25°C for 10 min followed by incubation at 50°C for 60 min. To finalize the reaction, the microtube was placed at 70°C for 10 min and cooled at 4°C. Control primers and GAPDH RNA were used as the positive control of cDNA synthesis reaction.

*Real-time PCR (RT-PCR)*

To achieve a maximum level of sensitivity the annealing temperature was optimized prior to RT-PCR by thermal gradient PCR. The gradient PCR reaction was done for SOX2, OCT4 and Nanog in the total volume of 20 µl Ampliqone (Taq DNA Polymerase Master Mix Red, Denmark) (Amplicon). In this process, 2μL (5-10pmols) template was used with 10µl amplicon, 1μL of each primer and sterilized DEPC water up to 20μL. The PCR products were run on 2% agarose gel to analyze the products after visualization.

In the next step, RT-PCR was performed using SYBR green master mix (Takara Biotechnology, China). The primers used for performing PCR and RT-PCR were as follows: for Sox2 F: 5′GACAGTTACGCGCACATGAA3′ and R: 5′TAGGTCTG CGAGCTGGTCAT3′; for OCT4 F: 5′GGTATTCAGCCAAACGA CCA3′ and R: 5′CACACTCGGACCACATCCTT3′; for NANOG F: 5′GTGATTTGTGGGCCTGA AGA3′ and R: 5′ACACAGCTGGGTGGAAGAGA3′; for GAPDH F: 5′AATCCC ATCACCATCTTCCA3′ and R: 5′TGGACTCCACGACATACTCA3′. Real-time PCR mixture contained 10 μl of SYBR green master mix, 1μl of forward and reverse primers and 2 μl of template DNA. The volume was adjusted to 20μl by DEPC water. The accuracy was ensured using the melting curve and gel electrophoresis (if required). GAPDH was used as the housekeeping gene in all reactions. RT-PCR was performed at least three times for each sample.

*Quantification of gene expression*

The cycle threshold (CT) method was used to quantify the level of gene expression. The relative difference was calculated using 2^-ΔΔCT^ formula where ΔCT was the CT of the target gene (or calibrator), which was subtracted from the CT value of the house-keeping gene. In the current study, GAPDH was considered as the housekeeping gene and the calibrator was the adjacent non-tumoral tissue. The CT values of test samples (tumoral tissues) were compared with the CT values of the control (non-tumoral adjacent tissue) samples. To assess the statistical significance between tumoral and non-tumoral adjacent tissues the REST software was used.

*Statistical analysis*

Using descriptive statistics, the mean, standard deviation, median and interquartile range were reported for quantitative variables and frequency as well as percentage were reported for qualitative variables. 

Non-parametric Kruskal Wallis and Mann Whitney tests were used to assess the correlation of OCT4, SOX2 and NANOG expression with the clinical and pathological parameters of patients. The association of expression of OCT4, SOX2, and NANOG with one another was analyzed using the Spearman’s correlation test. The data were statistically analyzed using SPSS version 22 (SPSS Inc., IL, USA) and P<0.05 was considered statistically significant. 

## Results

This study was conducted on 30 OSCC and 30 non-tumoral adjacent tissue specimens. The mean age of patients was 62.53±13.55 years. Tongue was the most common primary site of tumor (46.7%). Demographic and data regarding the staging of tumors and the pathological characteristics of OSCCs (pathological subtype, tumor grade, presence of necrosis and lymph node, vascular and perineural invasion) in patients were presented in Table 1. The majority of patients were in stage IV (53.4%) while there was no case with metastasis in patients at stage IV. 

*Expression of CSC markers in tumoral and non-tumoral tissues*

Expression of OCT4 (P=0.505) and NANOG (P=0.681) was not significantly different between tumoral and non-tumoral tissues. The results showed that the expression of SOX2 gene in the adjacent non-tumoral tissue was significantly higher than that in the tumoral tissue. Expression of SOX2 in the tumoral tissue was 0.169 times its expression in the adjacent non-tumoral tissue (P=0.049; Table 2). The association between SOX2, OCT4 and NANOG expression in the tumoral and adjacent non-tumoral tissues was evaluated and it was found that expression of SOX2 was significantly correlated with the expression of NANOG (Spearman’s correlation test, P=0.024, r=0.411).

*Association of expression of CSC markers with clinical and pathological characteristics of patients*

The expression of SOX2 was not significantly correlated with clinical or pathological characteristics of patients according to the Kruskal Wallis and Mann Whitney tests. Also, the expression of OCT4 and NANOG were not significantly correlated with clinical or pathological characteristics of patients (Table 3). 

**Table 1:** Demographic and Pathologic Information of OSCC Patients

Variable		No. of patients
Age	≤ 60 years	13
	> 60 years	17
Gender	Male	14
	Female	16
Ethnicity	Persian	8
	Middle East	14
	Azerbaijani	3
	Arab	2
	Unknown	3
Family History	Present	10
	Absent	20
	Tongue	14
Primary sit of tumor	Lips & Buccal Mucosa	7
	Gingivae	2
	Mouth Floor	3
	Parotid gland	1
	Unknown	3
Tumor Size (cm)	≤ 3	11
	3.1- 6	14
	> 6	5
T Status	T1	3
	T2	10
	T3	6
	T4	11
N Status	Nx	10
	N0	12
	N1	3
	N2	5
Metastasis	M0	30
	M1	0
TNM Stage	I	3
	II	7
	II	4
	IV	16
Pathologic Subtype	Keratinized	2
	Non-keratinized	28
Pathologic Grade	I	15
	II	13
	III	1
	Unknown	1
	Necrosis	2
	Lymphatic Invasion	4
	Vascular Invasion	6
	Perineural Invasion	8

**Table 2:** Comparison of the Expression of SOX2, OCT4 and NANOG in the Tumoral and Non-tumoral Tissues

Gene	Value	ΔCT of the tumoral tissue	ΔCT of the adjacent healthy tissue	P value*
SOX2	Mean± SD	5.55±5.08	2.99±4.80	0.049
	Median (interquartile range)	4.5 (1.14-9.09)	2.5 (1.17-7.0)	
OCT4	Mean± SD	3.30±5.22	2.45±4.55	0.505
	Median (interquartile range)	3.77 (0.05-7.23)	3.54 (0.13-5.73)	
NANOG	Mean± SD	5.45±4.75	4.91±5.40	0.681
	Median (interquartile range)	4.25 (1.86±9.39)	5.79 (2.71±7.99)	

*, T-test (REST software)

**Table 3:** Association of SOX2, OCT4 and NANOG with Clinical and Pathological Characteristics of Patients

	SOX2 expression			OCT4 expression		NANOG expression	
Variable	Classification	Median	P value	Median	P value	Median	P value
N	N0	16.0	0.636*	4.0	0.374*	7.81	0.572*
	N1	0.80		0.01		2.00	
	N2	0.03		0.60		0.50	
TNM	Stage I	5.31	0.367*	11.79	0.658*	7.31	0.399*
	Stage II	21.54		3.47		13.23	
	Stage III	0.80		0.01		2.00	
	Stage IV	0.01		0.39		0.37	
Histological grade	Grade I	0.27	0.458**	0.76	0.945**	0.16	0.146**
	Grade II /III	0.24		1.94		3.97	
Lymphatic invasion	Yes	0.53	0.803**	0.01	0.567**	2.0	0.471**
	No	0.01		1.90		4.02	

*, Kruskal Wallis test; **, Mann Whitney test

## Discussion

While the CSC theory has been proven in leukemia (Griffin and Lowenberg, 1986) and breast cancer (Aktas et al., 2009; Fillmore and Kuperwasser, 2008), studies on the role of CSC in the development and progression of oral cancer is still in their infancy. Several researchers have focused on studying the expression of CSC markers and their prognostic value in normal oral mucosa and OSCC. OCT4, SOX2, and NANOG, the core transcription factors known for their regulatory role in the pluripotency of embryonic stem cell, have been largely implicated as the marker of CSC in different malignancies (Chiou et al., 2008; Vaiphei et al., 2014). However, the oncogenic role and the clinical significance of these CSC genes have not been well investigated in the head and neck SCCs as well as OSCC. In the present study, we evaluated the expression of CSC markers of OCT4, SOX2, and NANOG in OSCC versus the adjacent non-tumoral tissue and showed significantly higher expression of SOX2 in the adjacent non-tumoral tissues compared to the tumoral tissue.

Carcinogenesis of oral cancer includes several stages, in which normal cells are progressively transformed to cancer cells. Stem and progenitor cells reside in tissue are the most common targets of genetic transformation and after going through several genetic mutations could convert to CSCs. Studies on the progressive development of different cancer have shown that in normal tissue adjacent to tumoral and preneoplastic tissues, the expression of CSC markers have increased that indicate the early molecular changes in normal tumor-adjacent cells and the possible role of CSCs in the process of carcinogenesis (Krishnamurthy and Nör, 2012). Therefore, measuring CSC markers could enhance the diagnosis accuracy of disease at the early stages and can be used as a prognostic indicator.

Sex-determining region Y-box 2 (SOX2) is a transcription factor involved in pluripotency and self-renewal of embryonic stem cells. In CSC, SOX-2 plays an essential role in the survival of malignant squamous cell and could protect CSCs against apoptosis (Hussenet and du Manoir, 2010).

Although our results showed increased expression of SOX2 in adjacent non-tumoral tissue, there are controversies regarding the expression of SOX2 and its clinical usefulness between different studies on OSCC. Qiao et al., (2014) showed the simultaneous expression of OCT4 and SOX2 in transforming oral mucosal samples in rat models as well as pre-neoplastic and neoplastic human tissue samples. Although the expression of these two markers in metastatic samples was lower than that in the primary tumor sites, their results showed no expression in normal tissues. In agreement with our results, Fu et al, studied 436 tumoral samples, 362 adjacent non-tumoral samples and 76 normal tissue samples (uvula) and revealed that the expression of SOX2 was higher in the adjacent non-tumoral tissue compared to the tumoral tissues (Fu et al., 2016). The same results were reported in other malignancies. Li et al. demonstrated that expression of SOX2 in the gastric mucosa and intestinal metaplasia was higher than that in stomach cancer cells (Li et al., 2004). Also, the high expression of SOX2 was found in normal bronchial mucosa compared to squamous dysplasia of the lungs (Yuan et al., 2010).

It has been suggested that increased expression of SOX2 may be involved in primary transformation and carcinogenesis of squamous tumors in the process of hyperplasia and dysplasia. Thus, increased expression of SOX2 in the non-tumoral adjacent tissues may indicate primary molecular changes similar to those occurring in preneoplastic tissues. Alternately, such a higher expression in the adjacent non-tumoral tissue may simply indicate the presence of normal squamous precursor cells with high expression of SOX2, which are more abundant in the adjacent non-tumoral tissue compared to the tumoral tissue (Fu et al., 2016).

Our results also showed no significant difference in the expression of OCT4 and NANOG between the OSCC and adjacent non-tumoral tissue. In contrast to our results, Vaiphei et al. study on esophageal SCC, showed increased expression of OCT4 in the esophageal mucosa adjacent to an area of mild dysplasia and basal hyperplasia (Vaiphei et al., 2014). In their study, normal mucosal biopsies were negative for OCT4.

Study the expression of SOX2, NANOG and OCT4 in our study showed that there existed no association between CSC markers and lymph node metastasis, tumor stage, pathological grade and lymphatic invasion. The significance of SOX2 in lymph node metastasis and distant metastasis has been previously evaluated and shown that higher expression of SOX2 is correlated with lymph node metastasis, distant metastasis and poorer prognosis of colorectal cancer (Li et al., 2015). Also, evidence has shown that SOX2 knockdown induces mesenchymal-epithelial transition, which is the reverse process of epithelial-mesenchymal transition. In the latter process, cells become polarized and mobile; the characteristic that is necessary for invasion and metastasis in epithelial malignancies. It has been shown that SOX2-positive tumors, maintain such characteristic during lymph node metastasis. This finding may further indicate that SOX2-positive tumors have higher capability and likelihood of lymph node metastasis. The expression of SOX2 at the site of lymph node metastasis compared to primary tumor site has been investigated in other malignancies (Michifuri et al., 2012). Accordingly, decreased expression of SOX2 is significantly associated with decreased angiogenesis and lymphangiogenesis in breast cancer (Chen et al., 2008). Another study on lung cancer indicated the association of SOX2 with lymphatic metastasis (Ren et al., 2016).

Studies on head and neck SCC also demonstrated a significant association between SOX2 expression and lymph node metastasis (Michifuri et al., 2012; Ren et al., 2016). However, there are few studies reporting that there is no link between SOX2 expression and tumor stage, lymphatic metastasis or distant metastasis in this type of SCC. Similar to head and neck SCC, the role of SOX2 in OSCC has not yet been fully elucidated. It has been demonstrated that SOX2 in oral cancer has two cytoplasmic staining patterns known as disseminated and peripheral. The disseminated pattern was significantly associated with metastasis (Nichols et al., 1998).

While Neumann et al. showed that higher expression of SOX2 at the site of primary tumor correlate with poorer prognosis and lymph node metastasis (Neumann et al., 2011), others demonstrated a significant correlation between increased expression of SOX2 and the absence of lymph node metastasis (Züllig et al., 2013). They suggested that the expression of SOX2 is a suitable indicator of the absence of regional lymph nodes metastasis in oral cancer. Using immunohistochemistry, Fu et al. evaluated the expression of OCT4, SOX2, and NANOG in oral cancer and showed that the expression of OCT4 and SOX2 increased with decreased tumor stage and size as well as the absence of lymph node metastasis (Fu et al., 2016). In our study, expression of OCT4, SOX2 and NANOG genes was slightly, but not significantly, higher in patients without lymph node metastasis.

To the best of our knowledge, this study is the first one to measure CSC markers at RNA level and that use of relatively large sample size is the strength point of our study. One limitation of our study was the absence of healthy tissue control for evaluating the expression of CSC markers, therefore, including normal sample is suggested for future studies. Studies on association of prognosis and survival rate with the expression of CSC markers could be an interesting topic for further researches in this line.

In conclusion,within the limitations of this study, the results showed that expression of SOX2 was significantly higher in the adjacent non-tumoral tissue compared to the tumoral tissue. The expression of OCT4 and NANOG was not significantly different between the tumoral and non-tumoral adjacent tissues. These findings suggest the possible use of SOX2 for early detection of OSCC. 
